# Alterations of anti-inflammatory lipids in plasma from women with chronic widespread pain - a case control study

**DOI:** 10.1186/s12944-017-0505-7

**Published:** 2017-06-12

**Authors:** Niclas Stensson, Bijar Ghafouri, Björn Gerdle, Nazdar Ghafouri

**Affiliations:** Pain and Rehabilitation Centre, Department of Medical and Health Sciences, Linköping University, Linköping, Sweden

**Keywords:** Chronic widespread pain, Inflammation, *N*-acylethanolamines, Palmitoylethanolamide, Oleoylethanolamide, Cytokines

## Abstract

**Background:**

Chronic widespread pain conditions (CWP) such as the pain associated with fibromyalgia syndrome (FMS) are significant health problems with unclear aetiology. Although CWP and FMS can alter both central and peripheral pain mechanisms, there are no validated markers for such alterations. Pro- and anti-inflammatory components of the immune system such as cytokines and endogenous lipid mediators could serve as systemic markers of alterations in chronic pain. Lipid mediators associated with anti-inflammatory qualities – e.g., oleoylethanolamide (OEA), palmitoylethanolamide (PEA), and stearoylethanolamide (SEA) – belong to *N*-acylethanolamines (NAEs). Previous studies have concluded that these lipid mediators may modulate pain and inflammation via the activation of peroxisome proliferator activating receptors (PPARs) and the activation of PPARs may regulate gene transcriptional factors that control the expression of distinct cytokines.

**Methods:**

This study investigates NAEs and cytokines in 17 women with CWP and 21 healthy controls. Plasma levels of the anti-inflammatory lipids OEA, PEA, and SEA, the pro-inflammatory cytokines TNF-α, IL-1β, IL-6, and IL-8, and the anti-inflammatory cytokine IL-10 were investigated. T-test of independent samples was used for group comparisons. Bivariate correlation analyses, and multivariate regression analysis were performed between lipids, cytokines, and pain intensity of the participants.

**Results:**

Significantly higher levels of OEA and PEA in plasma were found in CWP. No alterations in the levels of cytokines existed and no correlations between levels of lipids and cytokines were found.

**Conclusions:**

We conclude that altered levels of OEA and PEA might indicate the presence of systemic inflammation in CWP. In addition, we believe our findings contribute to the understanding of the biochemical mechanisms involved in chronic musculoskeletal pain.

## Background

Chronic widespread pain (CWP), which is often associated with fibromyalgia syndrome (FMS), is a significant health problem. The prevalence of CWP is approximately 10% [1, 2], and the prevalence of FMS is between 2 and 4% [3, 4]. The cause of CWP is multifactorial, but the underlying biochemical-neurobiological mechanisms are still unknown. However, both central and peripheral pain mechanisms influence the clinical presentation of CWP/FMS. Alterations in the “pain matrix” in the brain together with alterations in the descending control of nociception [5–9] suggest that CWP/FMS is a central hyperexcitability pain condition [8, 10, 11]. Some studies, however, have found that central alterations may be driven by peripheral alterations in muscles [3, 12] and/or in small fibres (A-delta and C-fibre) [13, 14].

Today, chronic pain is assessed using subjective and semi-objective methods. The lack of validated objective markers for involved pathophysiological nociceptive processes in chronic pain conditions makes diagnosis problematic, so treatments are often chosen based on a trial-and-error. However, emerging evidence suggests that a bidirectional crosstalk between nociceptor neurons and the immune system play a substantial role in the modulation of chronic pain [15–19], which makes molecular components of the immune system potential markers of persistent pain states.

Bioactive endogenous lipids – e.g., endocannabinoids (ECs) and *N*-acylethanolamines (NAEs) – are modulators of the immune system [20, 21] and influence pain and inflammation in the peripheral nervous system [22]. The NAE palmitoylethanolamide (PEA) activates peroxisome proliferator-activated receptor type-α (PPAR-α) [23], and exerts anti-inflammatory, analgesic, and neuroprotective actions [24]. Oleoylethanolamide (OEA) has anorexic properties [25] and regulates feeding and body weight through activation of PPAR-α [26]. OEA is also associated with both analgesic properties that may occur independently of PPAR-α activation [27] and with the induction of visceral pain via transient receptor potential vanilloid-1 activation [28]. Both PEA and OEA may modulate the excitability of peripheral nociceptors [22, 29]. Stearoylethanolamide (SEA) has been proposed to activate PPAR-γ [30] and to generate anti-inflammatory activity [31].

PPARs are nuclear receptors that are modulators of immune and inflammatory reactions [32]. They are involved in regulation of transcription factors and the expression of genes through a process called transrepression [33]*.* Both PPAR-α and -γ activation inhibit the transcriptional activity of nuclear factor kappa beta (NF-κB), the activator protein-1 (AP-1) [34, 35], and inflammatory gene expression [35, 36]. Activation of PPAR-α globally suppresses the interleukin-6 (IL-6)-stimulated acute phase response gene expression in mice [37]. PPAR-γ agonists blocks the production of tumor necrosis factor-α (TNF-α), IL-6, and interleukin-1β (IL-1β) [36]. A rheumatoid arthritis study found that IL-6 and interleukin-8 (IL-8) were down regulated in synovial fibroblasts and primary synoviocytes after treatment with OEA, PEA, and arachidonoylethanolamide [38]. In rats, systemic administration of OEA and PEA reduces plasma and brain levels of TNF-α-mRNA induced by lipopolysaccharides [39], and SEA suppresses serum levels of TNF-α by inhibiting NF-κB translocation, which may occur via PPAR-γ [30].

Cytokines are small non-structural proteins involved in modulation of the immune system and are generally classified as pro- or anti-inflammatory. Plasma and serum levels of cytokines have been investigated in respect to their association with CWP/FMS, but the results are inconclusive. High plasma levels of IL-8, IL-10, and TNF-α in FMS compared to controls have been reported [40]. When investigating serum levels of IL-6, IL-8, IL-10, and TNF-α, one study found significant higher levels of IL-10 in FMS but no alterations in the other investigated cytokines [41]. A meta-analysis concluded that plasma levels of IL-6 and IL-8 from patients with FMS were elevated [42].

We have previously reported significantly higher levels of NAEs in microdialysate samples collected from trapezius muscles in patients with CWP [43]. This study investigates systemic (plasma) levels of NAEs and cytokines in the same cohort. The primary aim was to investigate whether levels of anti-inflammatory lipid mediators OEA, PEA, and SEA, the pro-inflammatory cytokines TNF-α, IL-1β, IL-6, and IL-8, and the anti-inflammatory cytokine IL-10 differed significantly between healthy subjects and patients with CWP. Within this aim, correlations between levels of these substances and pain intensity were investigated.

## Methods

### Subjects

Results concerning NAEs in muscle tissue from the cohort investigated in this study (not exactly the same number) has previously been published [43, 44]. A total of 43 participants (19 CWP and 24 healthy controls (CON)) were recruited in the original study. Blood plasma of adequate quality (haemolytic plasma were excluded) were obtainable from 17 CWP and 21 CON and were included in this study. The CWP subjects were identified and recruited via a fibromyalgia patient organization and by reviewing the medical reports of former out-patients at the multidisciplinary Pain and Rehabilitation Centre, University Hospital, Linköping, Sweden. Inclusion criteria were female sex, age range 20–65 years, and widespread pain according to the American College of Rheumatology 1990 classification criteria [45]. Exclusion criteria were bursitis, disorders of the spine, tendonitis, capsulitis, postoperative conditions in neck/shoulder area, prior neck trauma, neurological disease, rheumatoid arthritis or any other systemic disease, metabolic disease, malignancy, severe psychiatric illness, pregnancy, and difficulties understanding the Swedish language. All participants were examined by a standardized and validated clinical examination of the upper extremeties according to Ohlsson et al. [46].

The CON subjects were recruited via advertisements in a local daily newspaper. Inclusion criteria were female sex, age range 20–65 years, and pain-free. The exclusion criteria were the same as the CWP group with the additional exclusion citerium of pain lasting more than 7 days during the past 12 months. Clinical examination was conducted as mentioned above.

### Pain intensity

The subjects were asked to estimate their whole body pain intensity on a 10-point numeric rating scale (NRS) with the following two end points: 0 = no pain and 10 = worst possible pain. The results concerning pain intensity have essentially (although not the same number of subjects) been published elsewhere [47].

### Blood sampling

Venous blood samples were drawn and centrifuged for 15 min (1500 g, 4 C°). Subsequently, the plasma was aliquoted (250 μL) in micro centrifuge tubes and stored at −70 °C.

### Measurements of NAEs

A liquid chromatography tandem mass spectrometry (LC-MS/MS) method was used to analyse NAEs in human plasma samples based on a previously published method [48]. Before measurements were taken, lipids were extracted from plasma following a previously described protocol [49]. Plasma samples were thawed and vortexed, and 25 μL of deuterated internal standard AEA-d4 (50 nM)) were added to each plasma and blank sample. Acetonitrile (ACN) (1200 μL) was added before vortexing and centrifugation (10,000 rpm, 5 min, 4 °C). Supernatants were added to 4.5 mL of milliQ-H_2_O containing 0.133% TFA (triflouro acetic acid). C8 Octyl SPE columns (6 mL, 200 mg) (Biotage; Uppsala, Sweden) were activated with 1-ml methanol and washed with 1-ml millQ-H_2_O before the samples were added. After washing with 1.5-ml ACN (20% with 0.1% TFA), the samples were eluted with 1.5-ml ACN (80% with 0.1% TFA), and dried by SpeedVacc. On the day of analysis, samples were reconstituted in 25 μL of LC mobile phase. The injection volume was 10 μL. All standards and internal standard were purchased from Cayman Chemicals (Ann Arbor, MI, USA).

We used an HPLC-MS/MS system containing a Thermo Scientific Accela AS auto sampler and Accela 1250 pump coupled to a Thermo Scientific TSQ Quantum Access max triple quadrupole mass spectrometer with an HESI II probe as ionization source. Liquid chromatography was performed using isocratic elution on a Xbridge C8 guard column (2.1 mm × 10 mm) coupled to a Xbridge C8 analytical column (2.1 mm × 150 mm) both with the particle size 2.5 μm obtained from Waters (Dublin, Ireland). The capillary temperature was set to 350 °C and sheath gas pressure to 40 arb units. The ion sweep gas pressure was set to 0.4 arb units. The selected reaction monitoring (SRM) (m/z) transitions 326.3/62.4, 300.3/62.4, 328.3/62.4 and 352.3/66.4 were used for OEA, PEA, SEA, and AEA-d4 respectively. The linearity of the measuring ranges was assessed with standard curves ranging from 5 to 1000 nM for all analytes in human plasma in triplicates and R^2^ ˃ 0.99 (± 0.01)) for all analytes. Isotopic dilution was used for quantification of the analytes, performed according to their area ratio of the deuterated internal standard signal area. Linear regression and equal weighting were applied. Xcalibur® (version 2.1, Thermo Scientific) software was used for peak integration and quantification.

### Measurements of cytokines

Plasma cytokines were analysed using two different immunoassays. TNF- α and IL-1β were analysed with the commercial High Sensitivity Human Cytokine Magnetic Bead Panel Immunoassay (MILLIPLEX® MAP for Luminex® xMAP1 Technology, EMD Millipore, Missouri, USA). The minimum detectable concentration (MinDC) was 0.7 pg/mL for TNF-α and 0.8 pg/mL for IL-1β. Standards, blanks, and samples were analysed in duplicates and mean values were used. IL-6, IL- 8, and IL-10 were analysed using multiplex proximity extension assay, (Proseek® Multiplex Inflammation I, Olink Bioscience, Uppsala, Sweden) per the manufacturer’s instructions [50, 51]. The unit is signal-to-background (dCq). The results from these three cytokines have been published elsewhere [52].

### Statistics

Traditional data analyses were perfomed using the IBM SPSS version 22.0 (IBM Corporation, Route 100 Somers, New York, USA) and the GraphPad Prism computer programmer version 6.03 (GraphPad Software Inc., San Diego, CA, USA). The number of patients needed to achieve sufficient power was based on the concentration of interstitial lactate of the trapezius in healthy controls and in patients with chronic trapezius myalgia reported in one of our previous studies [53]. Hence, using Power and Sample Size Calculation, ver. 3.0.229 based on the following parameters: a = 0.05, power = 0.8, difference between groups = 1.7, and SD = 1.7, it was found that 17 patients in each group were needed. Hence, this power analysis can be considered as post hoc calculation assuring that the number of subjects in the present study has the power to reveal relevant differences. T-test of independent samples was used for group comparisons. Bivariate correlation analyses (Pearson) was used, and a *p* ≤ 0.05 was used as level of significance in all analysis.

Traditional univariate and bivariate statistical methods can quantify level changes of individual substances but disregard interrelationships between them and thereby ignore system-wide aspects. Therefore, multivariate data analysis (MVDA) using the software SIMCA 14.0 (Umetrics, Umeå, Sweden) was applied as a complement to the traditional statistical methods. Principal component analysis was used for data overview. The SIMCA tools Hotelling’s T^2^ and distance to model in X-space were used to identify strong and moderate multivariate outliers, respectively. Orthogonal partial least squares-discriminant analysis was used for the multivariate regression analysis of group membership (CWP or CON) [54] using the levels of the investigated lipids, cytokines, and pain ratings.

For all MVDA analyses, data were log-transformed when needed using the auto transform function, and scaling to unit variance was applied [55, 56]. The parameters R^2^, Q^2^, and CV-ANOVA diagnostic were used to evaluate model quality. R^2^ describes the goodness of fit – the fraction of sum of squares of all the variables explained by a principal component [54] (R^2^ = 1 explains 100% of the data). Q^2^ describes the goodness of prediction – the fraction of the total variation of the variables that can be predicted by a principal component using CV methods. R^2^ should not be considerably higher than Q^2^. A difference greater than 0.2–0.3 implies overfitting, indicating poor robustness of the model is poor [55]. The CV-ANOVA diagnostic corresponds to a hypothesis test of the null hypothesis of equal cross-validated predictive residuals of the two compared models [57], measures the significance of the observed group separation, and returns a statistically significant *p*-value [55]. The variable influence on projection (VIP) indicates the relevance of each X-variable pooled over all dimensions and the Y-variables – the group of variables that best explains Y. VIP ≥ 1.0 was considered significant. Coefficients (OPLS scaled and centred regression coefficients) were used to note the direction of the relationship, positive or negative (in the text this is reported immediately after the VIP value).

## Results

### Background data

No significant group differences in age (years) (CWP: 41.7 ± 10 vs. CON: 47.9 ± 9.6) or body mass index (BMI) (kg/m^2^) (CWP: 24.2 ± 2.1 vs. CON: 26.8 ± 5.3) were found.

### Pain intensity

The pain intensity was significantly higher in CWP (5.3 ± 2.1) compared to controls (0.0 ± 0.0) (*p*-value ˂0.001).

### NAE concentrations

**Fig. 1:**
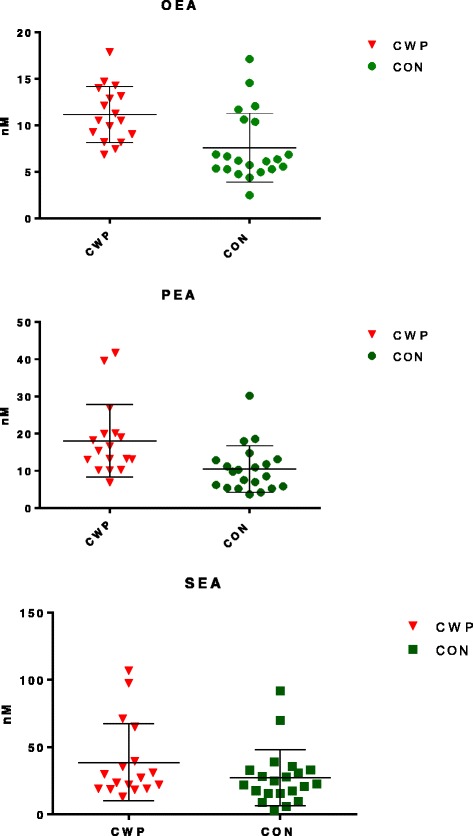
*Scatter plots* of concentrations (nM) in plasma for oleoylethanolamide (OEA), palmitoylethanolamide (PEA), and stearoylethanolamide (SEA) sampled from women with chronic wide spread pain (CWP) and healthy female controls (CON). The *horizontal lines* illustrate the group mean (*middle-line*) with *error bars* (± SD) above and below

Levels of NAEs were measured in all samples (Table 1). The levels and variation of NAEs are illustrated in Fig. 1. OEA and PEA levels were significantly higher in CWP than in CON. A tendency for higher SEA levels was found in CWP, but this trend did not reach statistical significance.

**Table 1:** Mean values and standard deviations for concentrations (*nM*) of oleoylethanolamide (*OEA*), palmitoylethanolamide (*PEA*), stearoylethanolamide (*SEA*) in subjects with chronic wide spread pain (*CWP*) and healthy controls (*CON*)

NAEs	CWP (*n* = 17)	CON (*n* = 21)	*p*-value
OEA	11.1 (3.0)	7.5 (3.7)	0.003^*^
PEA	18.1 (9.7)	10.5 (6.2)	0.006^*^
SEA	38.6 (28.7)	27.2 (20.7)	0.164

^*^indicates statistical significance

### Cytokine concentrations

The Luminex® assay measured TNF-α levels in all samples and IL-1β was measured in 21% of the samples (17 CWP; 20 CON). Levels under MinDC were substituted to MinDC/2. The Proseek® assay measured IL-6, IL-8, and IL-10 (presented elsewhere [52]) levels in all samples (16 CWP; 18 CON). No significant group differences in levels were found for the following cytokines: TNF- α (pg/mL) (CWP: 5.6 ± 1.7 vs. CON: 5.8 ± 3.6); IL-1β (pg/mL) (CWP: 0.6 ± 0.5 vs. CON: 2.1 ± 6.0), IL-6 (dCq) (CWP: 2.7 ± 1.7 vs. CON: 1.9 ± 1.5); IL-8 (dCq)) (CWP: 29.8 ± 25.8 vs. CON: 19.0 ± 5.9); and IL-10 (dCq) (CWP: 3.2 ± 1.0 vs. CON: 3.0 ± 0.6).

### Correlation analysis

**Fig. 2:**
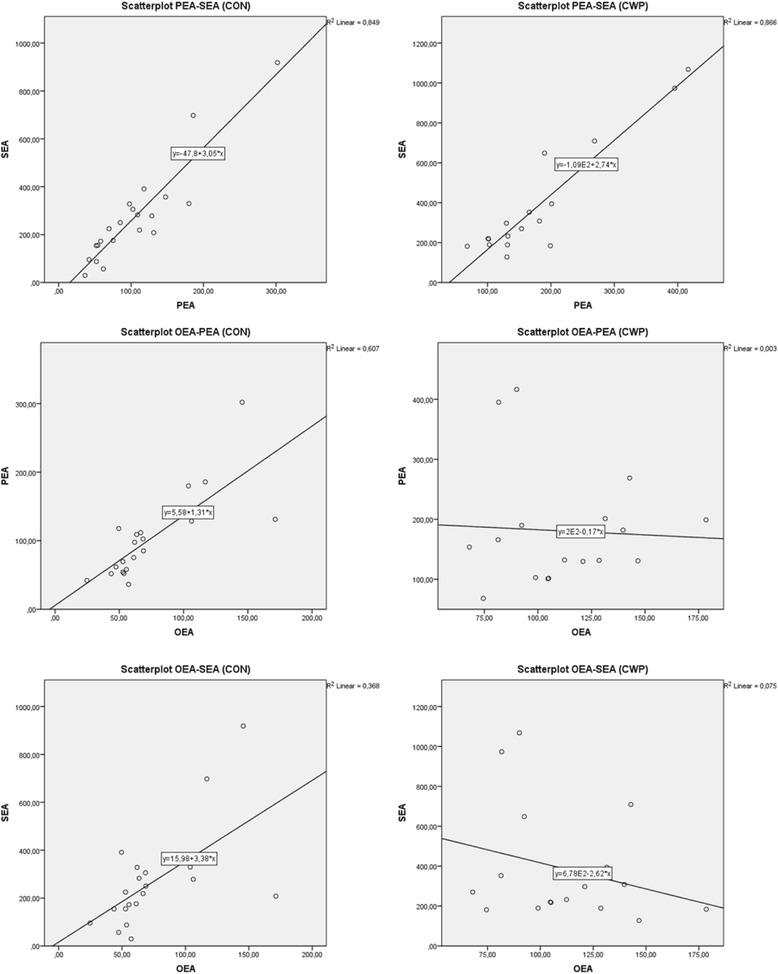
Bivariate correlation for the three *N*-acyletanolamines in the two groups illustrated with *scatterplots*. Best fitted regression (R^2^) *lines* describe the direction of the correlation. In the CON (*panel to the left*), the directions are consistently positive. In the CWP (*panel to the right*), the direction is positive for PEA vs. SEA but weakly negative for OEA vs. PEA and OEA vs. SEA

No correlation between NAEs and cytokines existed in the cohort as a whole or in the two groups analysed separately. When all participants (CWP + CON) were included, significant correlations existed between OEA and pain intensity (*r* = 0.49, *p* < 0.01). When this was the case, the two groups were tested separately. In CWP, a significant negative correlation existed between TNF-α and pain intensity (*r* = − 0.50, *p* < 0.05). Interestingly, significant inter-correlations existed between the lipids (OEA vs. PEA, *r* = 0.78, *p* < 0.01; OEA vs. SEA, *r* = 0.61 *p* < 0.01; PEA vs. SEA, (*r* = 0.92 *p* < 0.01) in CON, although in CWP a significant correlation only existed between PEA vs. SEA (*r* = 0.93, *p* < 0.01) – i.e., no correlation existed between OEA and PEA (*r* = −0.05) or OEA and SEA, (*r* = − 0.27). In Fig. 2, inter-correlations of the lipids are illustrated with scatterplots of the lipid levels in each group (CON, CWP). The linearity of each plot is illustrated by the best fitted regression line (R^2^) to the scatter.

### The relative importance of the lipids and cytokines - multivariate analyses

The data were primarily overviewed by a principal component analysis model to check for multivariate outliers – 38 observations (CWP and CON) and nine variables (NAEs, cytokines and pain intensity). No strong or moderate outlier was found by Hoteling’s T^2^ statistics (T^2^ Critical 99%) and distance to model in X-space (DCrit (0.05)).

In the following step, levels of NAEs, cytokines, and pain intensity were used for OPLS-DA regression of group membership. A strong significant regression model was obtained (R^2^ = 0.93, Q^2^ = 0.89, and CV-ANOVA: *P* < 0.001). As expected, pain intensity was the most important regressor, VIP = 1.80 (+). Interestingly, OEA and PEA were also important regressors, VIP = 1.19 (+) and 1.17 (+), respectively. Cytokines, SEA, BMI, and age had VIP ˂ 0.9 and were relatively unimportant regressors in that model.

**Fig. 3:**
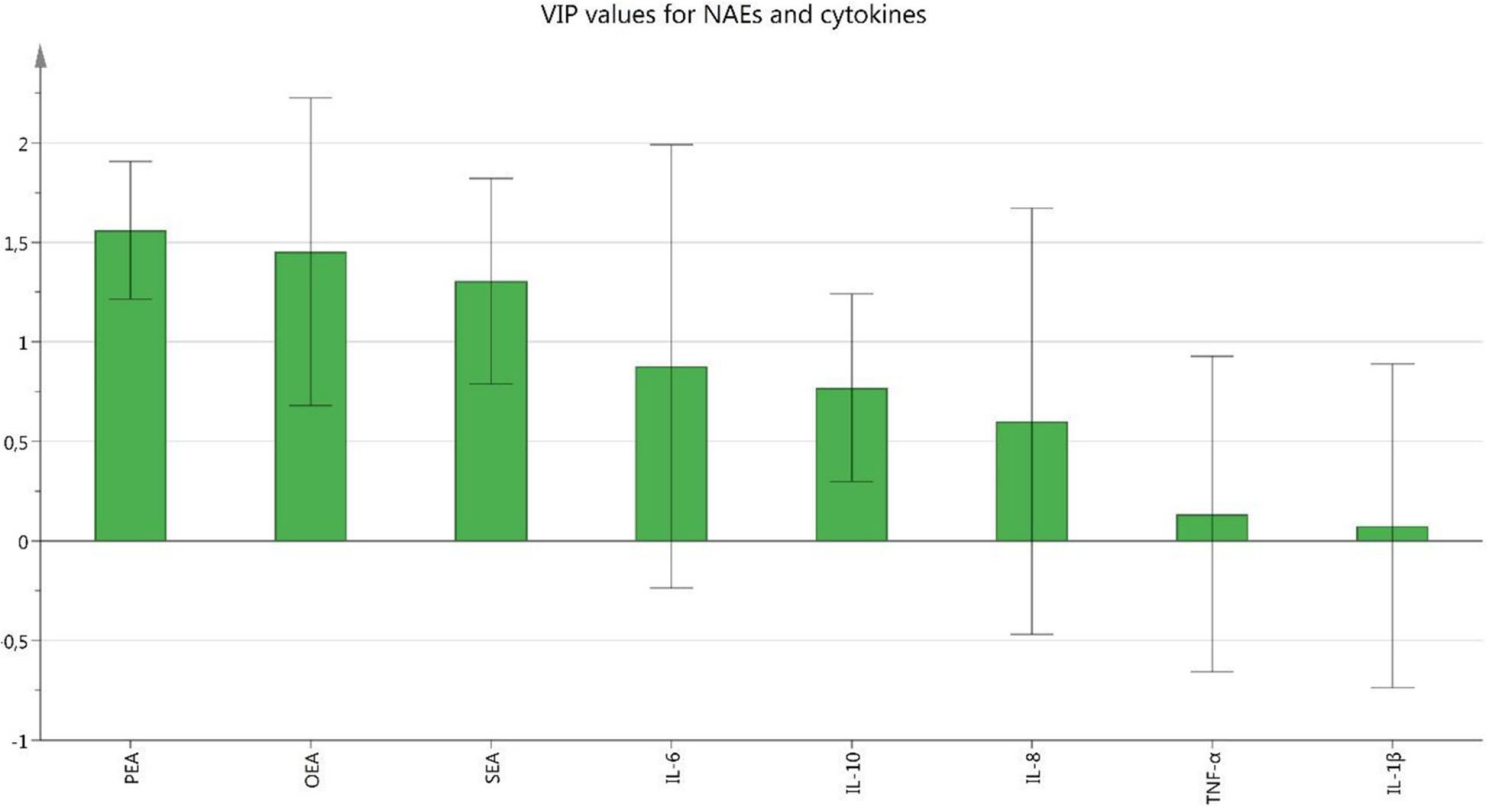
Variable importance of projection (VIP) values represented as *bars* with *error bars* of NAEs and cytokines. VIP > 1.0 is considered as significant. PEA, OEA, and SEA were the relatively strongest group-separating (CWP vs. CON) substances (VIP ≥ 1.30). The cytokines were relatively unimportant regressors in this context (VIP ≤ 0.86)

In the next step, only NAEs and Cytokines were used as regressors. A weaker yet significant model was obtained (R^2^ = 0.32, Q^2^ = 0.26, and CV-ANOVA: *P* = 0.012). Most important regressors in that model were PEA (VIP =1.57 (+)), OEA (VIP = 1.47 (+)), and SEA (VIP = 1.30 (+)). Hence, when inter-correlations between NAEs and cytokines were also taken into consideration (i.e., in contrast to the traditional statistical analyses), all three NAEs significantly contributed to the between group differentiation and thus were higher in CWP than CON. All cytokines had VIP ˂ 0.86, so they were relatively unimportant regressors compared to the NAEs with respect to group belonging. In Fig. 3, VIP values of all substances are represented as bars with error bars.

## Discussion

This study revealed three major findings.

- Significantly higher plasma levels of NAEs and especially for OEA and PEA were found in CWP.
- Multivariate regression modelling confirmed that cytokines were relatively unimportant compared to NAEs with respect to group belonging.
- No significant correlations existed between cytokines and NAEs.

A previous investigation of this cohort found that the CWP group had higher NAE levels in microdialysate locally sampled from the trapezius muscle during the first 2 h after microdialysis probe insertion [43]. These results indicate that levels of NAEs are altered in CWP although with some differences between microdialysate and plasma levels. In the microdialysate samples, SEA and OEA (not PEA) were significantly higher, but alterations of OEA and PEA (not SEA) were significantly higher in plasma. Our results taken together may reflect an altered NAE metabolism in CWP compared to CON.

To the best of our knowledge, there are only two previous studies that have investigated plasma levels of NAEs in CWP/FMS. High levels of the endocannabinoid arachidonoylethanolamide were reported in patients with FMS compared to controls [58]. Recently, Hellström et al. investigated the levels of OEA, PEA, SEA, and other related lipids in 15 woman with CWP and 27 healthy controls [59], but they did not find statistically significant group differences. A possible explanation for the different findings between their study and our study could be the selection of patients. Although both studies used the American College of Rheumatology CWP criteria from 1990, most patients (15/17) in ours were classified as FMS patients and had a NRS mean score of 5.3 ± 2.1 compared to a median NRS score of 3 for the CWP patients in Hellström et al.

In the present study, no alterations of traditionally investigated cytokines were found between groups, a finding that agrees with some reports and disagrees with other reports [40–42]. A significant negative correlation between TNF-α and pain intensity in CWP existed, which (to the best of our knowledge) has not been previously reported. Wang et al. found elevated levels of circulating TNF-α in FMS, but they did not observe any correlation between these levels and pain intensity [60]. In a more recent study, Christidis et al. did not find any difference between plasma levels of TNF-α between FMS and controls and did not find any correlation with pain intensity [61].

NAEs under investigation in this study have been proposed to suppress levels of various cytokines [30, 38, 39]. However, we found no correlations between cytokine and NAE levels. Another observation was that all NAEs positively inter-correlated significantly in CON. In CWP, a significant positive correlation existed only between PEA and SEA (Fig. 2). This difference in patterns between the two groups together with the group alterations indicates an altered NAE metabolism in CWP; however, the possible cause and relevance of such alteration needs more investigation. Our results concerning NAEs together with results from a panel of inflammatory proteins from the same cohort [52] and results from another cohort [62] may indicate that systemic inflammation is present in CWP.

By using MVDA, we confirmed the traditional statistical analyses. When all participants (CWP and CON) were included, significant models were obtained both with and without the pain intensity variable included. In those models, OEA and PEA were the most important (group separating) substances.

Because we already reported on elevated levels of NAEs in CWP from locally sampled trapezius muscles [43], the main strength of this study is that it measures systemic levels in the same cohort. The results confirm that systemic NAE levels are altered as well, which increases the data’s impact. Another strength of our study is that it compares the impact of cytokines potentially related to CWP with NAEs with respect to group belonging (CWP or CON). A majority of NAEs, including OEA, PEA, and SEA, have been reported to be influenced by diet [63, 64] and both PEA and OEA are associated with energy metabolism [65]. Therefore, one limitation of this study is that dietary factors were not captured. In addition, our study investigated a limited number of molecular components and our sample size was relatively small.

## Conclusions

The nociceptive and the immune systems are linked. The systems are specialized to prevent tissue damage and restore homeostasis by targeting and modulating harmful stimuli.

In this study, two classes of molecules associated with the nociceptive and immune systems were investigated (NAEs and cytokines) in CWP and in CON. Altered levels and altered relative composition of NAEs were found in CWP compared to CON. As partly reported elsewhere, no alterations of cytokine levels were found and no correlations existed between NAEs and cytokines. Circulating levels of NAEs may indicate systemic inflammation in CWP better than the investigated cytokines; however, more studies are needed.

## Abbreviations

CON: Healthy controls
CWP: Chronic widespread pain
FMS: Fibromyalgia syndrome
IL-10: Interleukin-10
IL-1β: Interleukin-1 beta
IL-6: Interleukin-6
IL-8: Interleukin-8
NAE: *N*-acylethanolamines
NRS: Numeric rating scale
OEA: Oleoylethanolamide
OPLS-DA: Orthogonal partial least squares-discriminant analysis
PEA: Palmitoylethanolamide
SEA: Stearoylethanolamide
TNF-α: Tumour necrosis factor-alpha
VIP: Variable influence on projection
